# Threat appeals in health communication: messages that elicit fear and enhance perceived efficacy positively impact on young male drivers

**DOI:** 10.1186/s12889-016-3227-2

**Published:** 2016-07-27

**Authors:** Rachel N. Carey, Kiran M. Sarma

**Affiliations:** 1 Department of Clinical, Educational and Health Psychology, University College London, 1-19 Torrington Place, London, WC1E 7HB UK; 2 School of Psychology, National University of Ireland, Galway, Newcastle Road, Galway, Ireland

**Keywords:** Health communications, Road safety, Driving behaviour, Perceived efficacy

## Abstract

**Background:**

Health communications often present graphic, threat-based representations of the potential consequences of health-risk behaviours. These ‘threat appeals’ feature prominently in public health campaigns, but their use is controversial, with studies investigating their efficacy reporting inconsistent, and often negative, findings. This research examined the impact of a threat-based road safety advertisement on the driving behaviour of young male drivers.

**Methods:**

To address limitations of previous research, we first identified a road safety advertisement that objectively and subjectively elicited fear using physiological and subjective measures. Study 1 (*n* = 62) examined the effect of this advertisement, combined with a manipulation designed to increase perceived efficacy, on speed choice. Study 2 (*n* = 81) investigated whether a state emotion, anger, impacts on the effectiveness of the advertisement in changing four distinct driving behaviours. Both studies examined short-term effects only.

**Results:**

Study 1 findings indicated that a high threat message, when combined with high perceived efficacy, can lead to a decrease in speed choice. Study 2 results suggested that increased levels of state anger may counteract the potential value of combining fear-arousing threats and efficacy-building messages.

**Conclusions:**

Findings suggest that threat-based road safety communications that target affective (fear) and cognitive (perceived efficacy) mechanisms can positively affect driving behaviours. State emotions, such as anger, may negatively impact on the effectiveness of the message. Taken together, these findings provide additional support for the use of efficacy-building messages in threat-based communications, but highlight the need for further research into the complex array of affective influences on driving.

**Electronic supplementary material:**

The online version of this article (doi:10.1186/s12889-016-3227-2) contains supplementary material, which is available to authorized users.

## Background

Road traffic collisions (RTCs) are responsible for approximately 1.2 million deaths per year and are the leading cause of mortality for young people [1, 2]. Young male drivers, in particular, are consistently overrepresented in global road fatality statistics [3–5]. Most RTC fatalities derive from human factors, including errors in decision-making and driver fatigue, and are considered to be preventable [6]. One of the key challenges facing public health practitioners and road safety agencies is that there is inconsistent empirical evidence on the mechanisms of change that can be targeted in road safety messages, which can positively impact on driving performance.

To date, road safety messages have typically been threat-based (termed ‘threat appeals’), presenting graphic depictions of the negative consequences that can result from risky driving [7] and ending with guidance on how to prevent collisions (e.g. reducing speed). Decades of inconsistent experimental findings, and evidence of defensive, maladaptive responses among certain audiences [8, 9], have shrouded the use of threat appeals in controversy [10]. While there is evidence that threat appeals can be highly effective when combined with content that enhances perceived efficacy [11], their potential to elicit negative effects, or no effect at all, means that their use is generally ‘ill-advised’ ([12], p.6). Despite this, threat appeals continue to play a prominent role in road safety advertisements, and their effectiveness in this context warrants further investigation. The studies reported in this paper seek to enhance our understanding of threat-based communications and their impact on behaviour. Specifically, we seek to identify the conditions, if any, under which threat appeals can have a positive impact on driving behaviours. Prior to presenting our findings, we first set the context for the research by providing an overview of important theoretical work in the area, and a brief synthesis of the general, as well as road safety-specific, threat appeals literature.

## Theoretical models applied to threat appeals

Given the importance of using theoretical frameworks to inform behaviour change strategies [13, 14], the current research was informed by a number of relevant theories. Theoretical models applied to threat appeal research include the fear-as-acquired drive model [15], the Parallel Response Model (PRM; [16]), the Health Belief Model (HBM; [17]), Protection Motivation Theory (PMT; [18]), the Theory of Planned Behaviour (TPB; [19]), Terror Management Theory (TMT; [20]) and the Extended Parallel Process Model (EPPM; [21]). We adopted an integrative approach, drawing on a number of theoretical perspectives and relevant empirical research. In particular, we focused on three key constructs. First, we examined self-efficacy (i.e. beliefs about one’s capability of performing a behaviour), which has been identified by many theories (e.g. HBM, PMT, TPB, EPPM) as a key determinant of behaviour. Second, we looked at fear arousal (i.e. the elicitation of fear following detection of a threat), which has a central role in influencing appraisals of threat (e.g. see PMT and EPPM), which in turn influence processing of a threat-based message. Finally, we investigated state anger, which has been highlighted by empirical research as having a negative impact on driving behaviour [22].

Although we were influenced by a number of relevant theories, given our focus on perceived efficacy and fear arousal, we drew in particular on the EPPM [22]. The EPPM was developed specifically as a framework through which to understand the range of complex psychological processes triggered in response to threatening messages. According to the EPPM, when a threat appeal is viewed by an audience, it is first appraised in terms of the perceived severity of the threat (i.e. the extent to which one believes the consequences of a road traffic collision are serious), and perceived susceptibility to the threat (i.e. the extent to which one believes one’s risk of being involved in a road traffic collision is high). If the threat is not perceived to be high, Witte argues, individuals are unlikely to process the message any further. If, however, the consequences of the depicted behaviour are perceived as serious and relevant, individuals next appraise the efficacy of a given recommended response. Perceived efficacy is made up of response efficacy (i.e. beliefs that the recommended response is effective in reducing the likelihood of an aversive outcome, e.g. ‘wearing my seatbelt is an effective way to avoid getting injured in a collision’) and self-efficacy (i.e. beliefs about one’s capability to carry out this recommended response, e.g. ‘I could easily wear my seatbelt and thereby reduce my chances of getting injured in a collision’).

Based on perceptions of efficacy, an individual may engage in one of two possible responses: danger control, or fear control. When a message is high in perceived threat, and perceived efficacy is high, danger control processes are initiated in which individuals are motivated to control the threat by adopting the recommended response. Contrastingly, when the threat is high, but perceived efficacy is low, fear control processes may be initiated in which the individual focuses on relieving the fear, rather than reducing the threat [23]. This can result in maladaptive behavioural responses such as denial (e.g. ‘I will never be in a road traffic collision’), or avoidance (e.g. ‘if I don’t think about it, it might go away’). Thus, high threat, high efficacy messages have the greatest potential for behaviour change [11, 21, 24].

Principles of the EPPM, particularly relating to the role of efficacy, have received substantial empirical support, with a number of reviews, updates, and commentaries highlighting its merit as a theoretical model [25–28]. However, an examination of the role played by perceived efficacy, fear arousal, and state anger in the context of driving behaviour is currently lacking.

## Threat appeal research

Psychological research into threat appeals dates back to 1953 [11]. During the early 1950s, the use of threat appeals was largely based on the then ‘common sense’ belief that the more fear the campaign evoked, the greater the chance the recommended behaviours would be carried out [29]. While some empirical research supported this position [30], findings across studies were inconsistent. In an early attempt to review and reconcile contradictory research findings from the period 1953–68, Higbee [29] argued that, despite researchers claiming that high threat appeals trigger defensive avoidance responses, making them less effective than lower threat messages, this was largely unsupported by the empirical evidence. He suggested that high threat messages are more effective than low threat messages, provided they include specific, easily implementable recommendations regarding the desirable health behaviour.

Subsequent to the period covered in Higbee’s review, a large number of experimental studies have been conducted, and researchers have identified particular conditions under which threat appeals are most likely to be effective. Witte and Allen’s [11] more recent review of the threat appeals literature (published in 2000) concluded that strong threat appeals with high levels of efficacy produce the greatest behaviour change. These conclusions have been supported by a number of subsequent empirical studies [31, 32].

Focusing specifically on the threat appeals literature relevant to road safety, a number of experimental studies have examined the impact of threat-based road safety advertisements on behavioural driving outcomes. Findings from these studies were synthesised in a meta-analysis by Carey, McDermott and Sarma [33]. Results of the meta-analysis indicated a large effect of threat appeals on fear arousal, but no evidence of any consistent impact on driving behaviour. In discussing their findings, the authors point to a number of conceptual and methodological limitations that exist within the experimental literature base.

First, the complexity of the relationship between threat (i.e. the content of the road safety message) and emotion (i.e. the response to the message) is not captured by a majority of studies. Second, there was an assumption within many threat appeal experiments that ‘high threat’ manipulations evoke fear in the audience, but this assumption was not generally supported with an objective manipulation check, and in general there was a lack of consistency in the way that fear was defined, measured and interpreted across the studies. Finally, studies largely failed to consider emotions, other than fear, that can be evoked by threat appeal messages. Threat appeals can elicit emotions such as guilt and shame, and researchers now recognise that the interplay between these different emotions can determine the effectiveness of the message [34–37]. Most notably, there is a need to distinguish between the related emotions of fear and disgust when examining responses to threat-based stimuli [38, 39].

There are two other key methodological limitations within the empirical driving literature. The first is a high level of variability across studies in the types of dependent variables employed. In a review of evaluations of anti-speeding messages, Plant, Reza and Irwin [40] found only one study meeting their inclusion criteria that included a direct (i.e. behavioural) measure of speed. The dependent variables used in threat appeal experiments in the context of road safety are largely self-report measures, such as ‘message acceptance’, a change in belief or attitude in line with the threat appeal message [41], anti-speeding attitude, or behavioural intentions. While self-report measures are useful and easy-to-administer, concerns have been raised about their validity in predicting driving behaviour [7].

The second issue is that laboratory-based experiments, while highly controlled, do not provide a representative indication of real-life driving scenarios and the variety of situational and intrapersonal variables that may determine message effectiveness and driving behaviours. For example, empirical research focusing on “the effect of affect” [42, 43] has highlighted the impact of state emotions on driving behaviour. While traditional approaches examining affective influences on driving have differentiated positive from negative emotions on a broad level, more recent research has demonstrated that different negative emotions (e.g. fear and anger) can have distinct effects on driving behaviours [44]. In particular, fear has been found to increase risk perception while anger reduces it [44]. Despite the implications of these findings, the conditions of heightened emotional arousal under which individuals often drive have not reliably been captured in a majority of experimental studies.

## The current investigation

The studies presented here test important aspects of the theoretical positions set out above, relating to the roles of perceived efficacy, fear arousal and state anger. The primary aim of this programme of research was to examine the conditions under which threat-based messages can be effective. First, we identified a road safety advertisement that objectively and subjectively elicited fear. The procedure and results are reported in summary below. This initial preparatory phase was important, as previous research has been criticised for failing to confirm that threat appeal manipulations actually evoke fear [33]. Our first study then examined the effect of a fear-evoking threat, when presented with a manipulation designed to increase efficacy, on a behavioural driving outcome. Our second study investigated whether state anger impacts on the effectiveness of the message. The research took place in Ireland and our sample focused on young male drivers, aged 18–24, based on research suggesting that this population take more risks on the road than any other driving cohort [3–5, 45].

## Identifying a fear-arousing threat appeal message

Prior to commencing the main body of research, we needed to identify a road safety advertisement that both objectively and subjectively elicited fear, which could then be used in subsequent studies. The methodology for this process is reported in summary here, but an extended report can be obtained from the authors.

Based on an initial focus group with 7 young male drivers, we selected 3 advertisements that were identified (via self-report) as evoking fear, and were rated as low in disgust. These three threat appeals (which we will refer to as ‘Ropes’, ‘Brakes’, and ‘Boy’; described briefly below) related to speeding behaviour, and all presented a physical threat (i.e. showed a collision) but differed in content and approach. Two of the threat appeal advertisements were taken from the UK’s *Think!* campaign, while a third was developed as part of the Drive SMART initiative by Surrey County Council. None had been aired on television in the Republic of Ireland, where this study took place.

The first message, ‘Ropes’, depicts the young male driver of a car beginning to accelerate, while ropes around his arms and legs become tighter and begin to cut him. The second message, ‘Brakes’, depicts a car travelling over the speed limit and hitting a person after failing to stop in time. The third message, ‘Boy’, depicts a boy walking around his old neighbourhood after being killed by a collision. There is a crash-impact moment in all three advertisements.

Sixty-one young males participated in a four-group experimental procedure, in which they were randomly assigned to one of three threat appeal groups, or a control group. We compared Heart-Rate (HR), Skin Conductance Level (SCL) and Facial Electromyography (EMG) between groups (i.e. exposed to different threat messages) and across time (i.e. at various points during the video exposure period). Researchers have emphasised the benefits of including physiological measures of both arousal (e.g. HR and SCL), and valence (e.g. Facial EMG) in studies of emotion, since we have different response systems to serve different adaptive functions [46, 47]. As such, the use of several distinct measures allows for additional detail and more representative information. Facial EMG represents a sensitive, and temporally specific, indicator of the valence of an emotional response, while HR and SCL data provide information on arousal.

Self-report measures of fear were also used, based on recommendations to measure fear through both subjective (i.e. self-report) and objective (i.e. physiological) methods. Self-reported fear was measured using both a one-item measure “How frightening did you find the last advertisement?” [48], with responses on a ten-point scale, from 'not at all frightening' to 'very frightening' and also by asking participants to indicate the extent to which they experienced six fear emotions: afraid, panicky, scared, worried, nervous and tense [49]. Participants watched three advertisements in total. All groups first watched a neutral advertisement, which provided ‘baseline’ data. The second advertisement contained the manipulation exposure (i.e. one of the three threat appeals or a control/neutral message), and therefore differed according to group. All groups then viewed a third, final, neutral advertisement.

Based on a protocol adopted in similar research [47], physiological data recordings were divided into ten second intervals. Within these intervals, average HR, SCL and Facial EMG responses, as well as peak activity, were identified. For the second advertisement (i.e. ‘Ad. 2’; the manipulation exposure), the 10-second interval surrounding the collision (the ‘impact point’; see [47]) was selected for analysis and compared to responses during baseline. For the three threat appeal advertisements, this ‘impact point’ fell within the final 20 s of the advertisements. The temporal equivalent within the neutral advertisement (presented to the control group) was chosen for comparison.

There were no statistically significant differences across groups at baseline for any physiological indices. Descriptive statistics for the HR data indicated there was a HR increase in all four groups from baseline to impact point. Individual group analyses indicated that this change was significant only for the Ropes group, who exhibited an increase of approximately 14 BPM (*M* = 13.91, *SD* = 15.33), *t*(13) = −3.40, *p* = .01, *d* = .86. For the EDA data, the Ropes group demonstrated a significant change from impact point to the end of Ad. 2, *t*(15) = 3.42, *p* < .01, *d* = .06, but there were no other significant differences. Facial EMG data indicated that, for the Ropes group, there was a significant increase in Corrugator muscle activity (a measure of negative affect) between baseline and impact point, *t*(15) = −3.19, *p* = .01, *d* = .53, and from baseline to the end of Ad. 2, *t*(15) = −4.54, *p* < .001, *d* = .67. Individual analyses of the other three groups did not reveal any significant changes over time.

For the subjective measure of fear, since the one-item measure was highly correlated with each of the six items, and since separate analyses yielded similar results, ANOVA and correlation analyses presented here are based on the one-item, ten-point measure. A one-way ANOVA revealed significant differences across groups, *F*(3,60) = 30.74, *p* < .001, partial η^2^ = .62, with the control group reporting the lowest levels of fear (*M =* 1.00; *SD =* .00), followed by the ‘Boy’ group (*M =* 4.00; *SD =* 1.96). The Brakes (*M =* 6.47; *SD =* 1.55) and Ropes (*M =* 5.31; *SD =* 2.12) groups reported high fear, and did not differ significantly from one another. Pearson’s correlation analyses were conducted across all groups together, and within each group individually. Results of the individual group analyses indicated that there were no statistically significant correlations between the one-item self-report fear measure and any of the physiological measures, for any of the threat appeal groups, during the impact point, contrary to findings of previous research [50].

The goal of this initial procedure was to identify an advertisement that was objectively and subjectively fear-arousing for young males. Findings point to the *Ropes* advertisement as evoking higher levels of arousal, and increased negative affect (i.e. a pattern consistent with fear arousal), compared to the other advertisements.

## Study 1

Study 1 examined the impact of threat and perceived efficacy on a behaviour-based driving outcome. Specifically, this experiment examined whether or not a threat appeal advertisement that is fear-arousing (i.e. ‘Ropes’) would lead to a corresponding adaptive change in a behavioural measure of driving (i.e. a reduction in speed). Speed was the focus of this first study as excessive speed is considered a critical road safety problem, and one that tends to be resistant to change [45]. For the purpose of this research, we use the term ‘speed choice’ to refer to participants’ responses in a video-based driving task, described in more detail below. Based on the EPPM [21], and following other research relating to perceived efficacy in this context [31, 51], we hypothesised that a manipulation designed to increase efficacy, when presented with the threat appeal message, would lead to a reduction in speed choice among young male drivers.

## Method

### Participants

*A-priori* power analyses estimated a sample of 112 participants, based on a large effect size. However, due to time constraints and difficulties recruiting this specific sample, we conducted preliminary analyses following data collection from 62 participants. We found significant differences across groups (with a medium effect size) with this sample. The 62 participants were male, aged 18–24 (*M* = 20.92, *SD* = 3.21) and in possession of a full driver’s licence (90 %, *n* = 56), or a provisional/novice driver’s licence with a minimum of one year’s driving experience (10 %, *n =* 6). Of the sample, 79 % (*n =* 49) were university students.

### Design

The study adopted a four-group experimental design and compared speed across time and between groups. The independent variable was message type. Group 1 were exposed to a threat appeal message (threat only group). Group 2 were exposed to a threat appeal along with an efficacy manipulation (threat + efficacy group). Group 3 were exposed to a neutral advertisement and to ‘neutral’ cognition prompts, described below (neutral cognitions group), and Group 4 were exposed to a neutral message only (control group). We hypothesised that the threat appeal advertisement would lead to a reduction in speed (i.e. a main effect for group), but that this effect would only occur when, consistent with the EPPM, perceived threat and efficacy were high. The dependent variable was participants’ response to an interactive video-based speed task, in line with calls for the use of behaviour-based outcomes in this type of research [26, 52].

### Advertisements

Each group was exposed to three advertisements (Distractor – Manipulation Exposure – Distractor). The distractor advertisements were used to obscure the true nature of the experiment, and were instructional videos about car maintenance and repair. For Group 1 (threat only), the exposure advertisement was the Ropes road safety advertisement. This advertisement, which was found to elicit subjectively and objectively measured fear among young male drivers, depicts a young man driving a car, while other young people in the back and passenger seats urge him to go faster. As the man accelerates, ropes around his arms and legs become tighter and begin to cut him, at which point there is a sudden, brief, crash-impact moment. The video then cuts to a scene where the young man is sitting in a wheelchair, while his friend sits crying beside him. Thus, while this advertisement contains a clear physical threat, it also taps into normative influences, including peer pressure. We edited the video such that the final screen simply displayed the words “Don’t Speed”.

Group 2 (threat + efficacy group) viewed the same exposure as the threat only group. However, for this group, four questions appeared at the bottom of the screen during the *Ropes* advertisement that encouraged participants to engage with the content of the advertisement (e.g. “Imagine you are the person driving the car, how might you react?”). Further, following the “Don’t Speed” screen, this group were presented with a number of questions that appeared on separate screens for ten seconds each (e.g. “What kind of strategies could you employ to avoid being involved in a road traffic collision?”). The aim of these questions was to increase perceived threat and efficacy among this group.

Group 3 (neutral cognitions group) viewed a neutral advertisement, which was an instructional video about air conditioning systems in cars. Questions such as “How useful do you think this video is?” appeared at the bottom of the screen, in order to test whether probing general cognitions about a driving-related video would affect behavioural responses. Finally, Group 4 (control group) viewed the same neutral advertisement as Group 3, but did not see any questions on screen.

### Measures

The four constructs of interest (i.e. perceived self/response efficacy, severity and susceptibility) were each measured using single items [31], with participants responding on a 7-point scale. Perceived severity was measured using the item “Thinking about a road traffic collision, I believe the consequences are…”, where scale response options ranged from 1 (= *Not at all severe*) to 7 (= *Very severe*). Perceived susceptibility was measured using the item “I believe my chances of getting involved in a road traffic collision are…” with participants indicating their response on a scale from 1 (= *Not at all likely*) to 7 (= *Very likely*).

Perceived self-efficacy, in relation to the recommended behaviour (i.e. reducing speed), was measured using the item “Thinking about ways I could change my behaviour that would reduce my chances of being involved in a road traffic collision (e.g. driving more slowly), I believe changing my behaviour in this way would be…” [Response scale: 1 (= *Very easy*) to 7 (= *Very difficult*)]. Perceived response efficacy, in relation to the recommended behaviour, was measured using the item “Thinking about the effectiveness of a change in behaviour (e.g. driving more slowly) in reducing my chances of being involved in a road traffic collision, I believe this kind of a change in behaviour would be…” [Response scale: 1 (= *Not at all effective*) to 7 (= *Very effective*)]. One of the items (self-efficacy) was reverse-scored, and each of the 4 items was analysed separately.

#### Driving scales

Participants were asked to complete a number of self-report driving scales. The Speeding and Rule Violation (SRV) subscale of the Driver Behaviour Scale [53] was used, and contains six items (e.g. “Overtake the car in front, even when it keeps the appropriate speed”) that measure the frequency with which participants engage in SRV, from 1 (= *Never*) to 5 (= *Very Often*). Items were summed to a scale total, with higher scores indicating higher levels of SRV. Internal consistency for this scale was acceptable (α = 0.83).

The short-form of the Driving Anger Scale [54] was used to measure participants’ tendency to become angry while driving. The scale includes fourteen items that describe various scenarios in which an individual may be likely to become angry (e.g. “Someone backs right out in front of you, without looking”). Participants were asked to respond to these statements by indicating the amount of anger that would be provoked, from 1 (= *none at all*) to 5 (= *a lot*). Items were summed to a scale total (α = 0.75).

#### Additional measures

A number of additional scales were included during data collection for Studies 1 and 2, which are not discussed in this paper, but which we list briefly here. At the end of the study, participants were asked to complete an extended self-reported fear measure [49], a measure of death thought accessibility [55], and a number of additional driving-related scales that included questions relating to their driving history (i.e. history of road traffic collisions, driving offences, incidence of racing another driver on a public road). These measures were all presented to participants following completion of the driving behaviour measure. This ensured that the measures did not impact on the dependent variable (i.e. have priming effects).

### Dependent variable: driving behaviour measure

Driving behaviour was measured using an Irish version of the Video Speed Test (VST), based on a driving measure that has been used and validated in previous research [56–59]. The VST involves the presentation of simulated driving scenarios through digital video images.

We developed this measure by filming driving scenes, from the driver’s perspective, on motorways (3 scenes) and dual carriageways (6 scenes). During filming, the vehicle speed was kept constant at the speed limit (i.e. 100 kph on the Dual Carriageway and 120 kph on the motorway). The video clips were edited such that the speed of the vehicle from which the footage was shot appeared to increase incrementally. These speed increases were created during the editing process, as opposed to during filming, as it allowed for more consistent, regulated and specific speed increases. Six edited clips were chosen for use, each of which was approximately one minute in duration. The clips were projected from a Hitachi CP-X301 data projector, and the resulting image was 1470 (width) × 830 (height) millimetres.

A pedal was attached to an Ergodex DX1 board, which was connected to the P.C. via a USB port, making it an interactive part of the driving task. Participants were asked to press down on the ‘accelerator’ pedal to begin the driving scene, and were instructed to lift their foot fully off the pedal once the car had reached ‘the speed at which they would normally drive’. When participants lifted their foot off the pedal, the driving scene ended, and their response-time was recorded in milliseconds. Six driving scenes were completed at baseline, and then again post-exposure. Participants’ mean response time across the scenes was recorded for pre-and post-manipulation, and change scores were calculated (see below). The dependent variable for this study, therefore, was the change in response time (i.e. in milliseconds) from pre- to post-manipulation.

### Procedure

Participants first completed a trial version of the driving task, containing three scenes (not used in the analysis), in order to become familiar with the VST, pedal and procedure. They then viewed and responded to the six driving scenes, pre-manipulation, as a baseline measure of speed. Following this, they were presented with two simple distractor tasks. Participants were then randomly assigned to one of the four groups (i.e. threat + efficacy, threat only, neutral cognitions and control), and were presented with the advertisements as described above (see Additional file 1 for an overview of the Study 1 experimental procedure). After the videos were over, participants were asked to complete the driving task once more, ostensibly 'in order to collect as much data as possible from each participant'. Once the driving task had been completed for the second time, participants were presented with one final questionnaire, measuring cognitive responses, driving anger, SRV, and the other scales listed above.

## Results

### Calculating speed change

A one-way ANOVA conducted on the VST data indicated that there were no significant differences across groups at baseline. Change scores were calculated for each participant by subtracting their baseline response time from that at post-manipulation. Positive change scores indicate greater response latency (i.e. higher speed at post-manipulation), while negative change scores indicate an earlier response press (i.e. lower speed at post-manipulation), since the speed of the car increased as the clip progressed.

### Perceived threat and efficacy

A one-way ANOVA was first conducted to examine differences across groups in terms of perceived severity, susceptibility, response efficacy and self-efficacy. This was carried out in order to examine whether (i) perceived threat was higher among participants who viewed the threat appeal, compared to those who viewed a neutral video and (ii) perceived efficacy was higher among participants exposed to both the threat appeal and the efficacy manipulation, compared to all other groups.

The data for perceived severity, susceptibility, response efficacy and self-efficacy appeared to be negatively skewed, with skew values exceeding acceptable levels (Kolmogorov-Smirnov [K-S] test result *p* < .05 for all cognitions), so the values were reflected (i.e. each score was subtracted from the highest score + 1), and subjected to a log transform. This reduced Skewness to within acceptable levels (i.e. not greater than twice the standard error). ANOVA results for the four groups presented here are based on these log-transformed data.

A one-way ANOVA revealed significant main effects for each of the four cognitions [Severity, *F* (3, 61) = 3.09, *p =* .04, partial η^2^ = .14; Susceptibility, *F* (3, 61) = 5.01, *p* < .001, partial η^2^ = .21; Self-efficacy, *F* (3, 61) = 7.95, *p* < .001, partial η^2^ = .29; Response efficacy, *F*(3, 61) = 3.50, *p =* .02, partial η^2^ = .15]. *Post-hoc* Tukey tests suggested that, for perceived severity, the threat + efficacy group differed significantly from the neutral cognitions group (*p =* .04), but no other significant between-group differences emerged. For perceived susceptibility, the threat + efficacy group and the threat only group differed significantly from the control group (*p* = .01 and .04, respectively), but not from the neutral cognitions group. For self-efficacy, the threat + efficacy group differed significantly from the threat only (*p* < .001), neutral cognitions (*p =* .01), and control (*p* < .001) groups. Finally, the threat + efficacy group differed significantly from the control group in terms of response efficacy (*p =* .01), but did not differ significantly from the other groups.

### Hypothesis testing

**Fig. 1:**
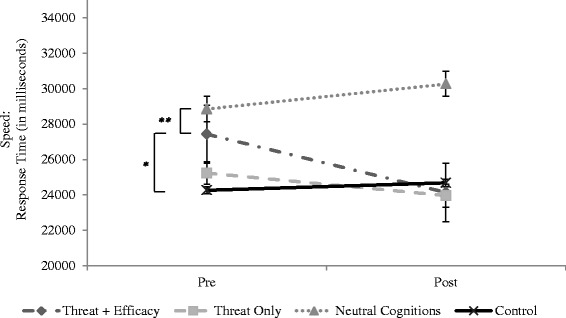
Change in Speed from Baseline to Post-manipulation in Study 1, across four groups. Asterisks denote significance between groups (difference between threat + efficacy and neutral cognitions, and difference between threat + efficacy and control). * *p* < .05, ** *p* < .01

Study 1 hypothesised that a manipulation designed to increase efficacy, when presented with a threat appeal message, would lead to a reduction in speed choice. Descriptive statistics (see Table 1 and Fig. 1) suggested that the most notable change in speed was among the threat + efficacy group, whose response press at post-manipulation (on average across all scenes) was approximately three seconds earlier (*M = −*3286.92, *SD =* 3388.33) than at baseline. Results of a one-way ANOVA indicated a significant main effect for group (*F* (3, 61) = .6.60, *p* < .001, partial η^2^ = .26).

**Table 1:** Descriptive Statistics (in Milliseconds) for Speed Choice Pre- and Post-Manipulation (Study 1)

	Pre	Post
Group	Mean (SD)	Mean (SD)
Threat + Efficacy	27428.75 (8194.71)	24141.83 (6333.25)
Threat Only	25236.33 (8936.88)	23958.95 (8695.39)
Neutral Cognitions	28847.34 (7043.26)	30263.10 (5749.29)
Control	24265.15 (9463.26)	24680.73 (8586.40)

*Post-hoc* Tukey test procedures suggested that the threat + efficacy group changed their speed significantly more than the neutral cognitions group (*p* < .001) and the control group (*p =* .01). Contrary to expectation, there was no significant difference between the threat + efficacy and the threat only groups (*p* = .29). However, unlike the threat + efficacy group, the threat only group did not significantly differ from the control group or the neutral cognitions group. There were no other significant differences across groups.

Correlational analyses indicated that driving anger (i.e. tendency to become angry while driving) was negatively correlated with perceived severity (*r = −*.31, *p =* .02), and response efficacy (*r = −*.28, *p =* .03), and positively correlated with SRV (*r =* .46, *p* < .001) and baseline driving speed (*r =* .26, *p =* .04).

## Discussion

In this study, participants who viewed an exposure designed to increase perceived threat (i.e. severity and susceptibility) and efficacy (i.e. self-efficacy and response efficacy) reported higher levels of these cognitions than all other groups, and reduced their speed significantly more than those in the neutral cognitions and control groups. Although there was no significant difference between the threat + efficacy group and the threat only group, the threat only group did not significantly differ from the control group or the neutral cognitions group. Thus, the data provide support for our hypothesis that the threat appeal would lead to a reduction in speed when perceptions of efficacy were high. These findings are consistent with experimental findings from the driving literature [31, 32].

Although the threat appeal message, for both threat groups, ended in a screen reading ‘Don’t Speed’, high perceptions of efficacy and significant reductions in speed were evident only among the group who received the additional efficacy manipulation. This suggests that road safety messages may achieve better impact by incorporating multiple efficacy recommendations. However, the specific efficacy messages that should be harnessed in such campaigns is unclear and merits further research. As noted by Tay [60], while it is relatively straightforward to provide the audience with an effective, achievable strategy for avoiding drink-driving (i.e. taking a taxi, nominating a designated driver) or fatigued driving (i.e. pulling the vehicle over and drinking a caffeinated beverage), few such explicit strategies exist in relation to speeding.

Since response efficacy is widely considered one of the most important characteristics in message persuasiveness [61] new, creative approaches to increasing efficacy in experimental studies are increasingly being called for. The approach adopted here provided a subjective efficacy manipulation that can be easily applied to other contexts. It is important to note, however, that since we did not have an ‘efficacy-only’ condition, we were unable to test interaction effects in a factorial design.

Correlational data analyses point to driving anger as being a potential variable of interest for studies in this area. Participants who were higher in driving anger were also higher in SRV, chose higher driving speeds at baseline and had lower perceptions of severity and efficacy.

Study 1 advanced on previous studies by including a behaviour-based dependent variable, and by using a manipulation that has been systematically identified as eliciting a fear response. However, since the outcome measure in this study related only to speed choice, questions remained regarding the effect of the threat appeal message on other driving behaviours. Further, given the link between anger and speed choice identified in this study, and in previous research, we felt it important to explore the impact of state anger on driving. This is particularly important given that state anger may be inadvertently evoked by emotive road safety messages – as well as during challenging driving conditions.

## Study 2

Previous research suggests that the psychosocial factors implicated in risky driving vary across different types of behaviours [60, 62], for example, speeding as compared to close following (i.e. tailgating). Study 2 examined the impact of a threat appeal, found to lead to a decrease in speed, on three additional driving behaviours. Study 2 also addressed the lack of research on the potential for state emotions to influence the relationship between persuasive communication and driving behaviour. Despite evidence of the effects of anger on driving [22, 63–65], there is very little research focusing on the impact of anger on individuals’ responses to threat-based road safety advertisements.

We hypothesised that participants exposed to a threat + efficacy manipulation (as in Study 1) would exhibit a change in driving behaviours (i.e. a reduction in speed choice, following distance, gap acceptance and overtaking), relative to baseline, and compared to all other groups. Second, we hypothesised that participants exposed to an anger-provoking manipulation, who are not exposed to the threat appeal or efficacy manipulation, would exhibit increased speed choice, following distance, gap acceptance and overtaking relative to baseline, and compared to the other groups.

## Method

### Participants

Eighty-one participants took part in this study. All participants were male, aged 18–24 (*M* = 19.80, *SD* = 1.84), and in possession of a full driver’s licence (77.8 %, *n* = 63), or a provisional driver’s licence, with a minimum of one year’s driving experience (22.2 %, *n =* 18). Of the sample, 87.7 % (*n =* 71) were university students.

### Design

The study adopted a four-group experimental design, with participants randomly assigned to conditions. Group 1 were exposed to a threat appeal with efficacy manipulation (threat + efficacy group; as in Study 1), Group 2 were exposed to a threat appeal with efficacy manipulation with additional anger manipulation (threat + efficacy + anger group), Group 3 were exposed to a neutral message plus the anger manipulation (anger only group), and Group 4 were a control group. Four simulated driving behaviours were used as dependent variables – speed, following distance, gap acceptance, and overtaking [56, 58, 66, 67].

### Materials

#### Advertisements

As in Study 1, each group was exposed to three advertisements (Distractor - Manipulation Exposure – Distractor), where the middle advertisement differed across groups. The distractor advertisements were the same as those used in the previous study, and again served to obscure the true nature of the experiment. The threat appeal message used in this study was the same high-threat road safety advertisement as that used in Study 1 (i.e. *Ropes*). Given that the threat + efficacy manipulation effectively reduced speed in Study 1, we did not include a ‘threat only’ group in this study; all groups who saw the threat appeal were also exposed to the efficacy manipulation, as in Study 1. Two of the four groups watched the threat appeal message with the efficacy manipulation, and one of these groups also received an anger manipulation. The two other groups viewed a neutral video, which was the same as that used in Study 1.

#### State anger

The anger manipulation used in the current study was an event-recall task. Participants in the two anger groups were asked to answer two open-ended questions. The first question required participants to “describe 3–5 things that make you most angry”, and the second question asked that participants “describe, in detail, the one situation that makes you, or has made you, the most angry you have been in your life”. This is a mood induction that is subjective to the individual, and has been used to elicit anger in a number of previous studies e.g. [68, 69]. Participants in the two ‘no-anger’ groups were asked to describe the experience of watching television.

Since labelling emotions has been found to reduce their salience [70], a manipulation check was not used. Rather, the anger task was first piloted (as in [68]) with 19 males, aged 18–24. Participants in the pilot were randomly allocated to the anger task (anger condition; *n =* 10), or to the neutral task (control condition; *n =* 9), and were then asked to rate the extent to which they felt 16 emotions: anxious, angry, amused, disgusted, downhearted, engaged, fearful, frustrated, happy, interested, irritated, nervous, mad, repulsed, sad, joyful [68, 71]. The ‘angry’ and ‘mad’ items were averaged to give a composite measure of anger. An independent samples *t-*test was conducted to examine differences across groups, and revealed that those who received the anger induction reported higher levels of anger (*M =* 4.15, *SD =* 1.33), than those in the neutral condition (*M =* 2.39, *SD =* 1.65; *t* (17) = −2.57, *p* = .02) and the magnitude of this difference was large (*d =* 1.17).

#### Driving behaviour measures

We developed four driving behaviour measures for the purposes of this study: speed (SPD), following distance (FOL), gap acceptance (GAP), and overtaking (OTK). As with the VST, described earlier, we developed these additional measures by filming driving scenes from the driver’s perspective. Participants completed trial versions of all tasks before beginning. For the SPD task (measured using our VST, described earlier), participants completed four scenes (two motorway scenes and two dual-carriageway scenes) at baseline, and then again post-exposure. We reduced the number of scenes from 6 (as in Study 1) to 4 to reduce the burden on participants. For the FOL task [67], participants viewed two scenes in which the car from which the footage was shot gradually approached the back of another car on a motorway. They were asked to press down on the response pedal to begin the driving scene, and were instructed that they would need to make two responses. First, they were asked to lift their foot fully off the pedal once the car had reached their ‘normal following distance’ (Normal FOL), the “distance at which they would normally follow another car”. Second, when participants reached a following distance at which they felt “dangerously or uncomfortably” close (Dangerous FOL), they were asked to press the Space bar on the keyboard (labelled ‘Brake’). Responses were measured in milliseconds from the start of the scene to Normal FOL, and to Dangerous FOL. The two Normal FOL (i.e. pedal) and Dangerous FOL (i.e. brake) responses for the trials were averaged. Higher scores indicate greater response latencies (i.e. closer following distance).

For the GAP task [67], participants viewed scenes in which the car from which the footage was shot was at a T-junction, attempting to turn left on to a busy main road (note that, in Ireland, vehicles drive on the left side of the road). The scene is shot from the perspective of the driver, with the camera positioned on the right-hand window (i.e. focusing on the oncoming traffic). Participants were instructed to monitor oncoming traffic, and to press the response pedal once every time there was a gap between cars that they would be willing to use to join the flow of traffic. Participants completed this task for two scenes, comprised of 22 gaps, many of which were unsafe (i.e. another car was coming very closely and/or quickly). A GAP score was calculated for participants by summing the number of gaps they selected (i.e. the number of pedal responses), with higher scores indicating more risky gap selections.

Finally, the OTK task involved participants viewing three videos containing six possible overtakes, in which the camera car was following a lead vehicle on a primary road (i.e. roads forming major routes between urban areas). The footage paused at various moments during the video, and participants were instructed that they should press the response pedal if they would overtake the car in front at the point indicated. A similar type of overtaking task has been adopted in previous research [66]. The number of pedal presses made by participants, over these three scenes, was recorded, and a total overtaking score was calculated per participant. Higher numbers of responses indicated greater levels of overtaking.

### Procedure

Participants completed each of the tasks in order to provide baseline driving data, and were then presented with the three videos. After the exposure period was over, all participants were asked to complete a distractor (word-search) task. Following this, the two anger groups were given the anger-recall task, while the two no-anger groups were given the neutral description task (see Additional file 2 for an overview of the Study 2 experimental procedure). Participants were then asked to sit once more in front of the projector, and to complete the four driving tasks again. They were then presented with one final questionnaire, measuring perceived threat and efficacy, driving anger, trait anger, driving history, and SRV. Participants were also asked the question ‘Do you believe you know what the purpose of today’s study is (if yes, please explain)?’ In response, 88 % of participants answered ‘yes’, however, of these, less than 5 % gave a correct answer. Responses (coded as 1 = no, 2 = yes-correct and 3 = yes-incorrect) did not differ significantly between groups.

## Results

### Calculating the dependent variables

Results indicated that there were no significant differences across groups at baseline for SPD, Normal FOL, Dangerous FOL, GAP or OTK. Change statistics were calculated for each participant for each of the four tasks.

### Hypothesis testing

**Fig. 2:**
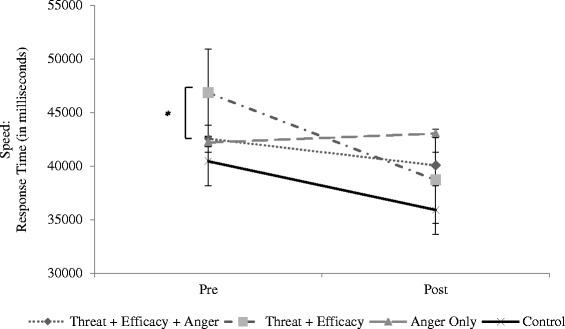
Change in Speed from Baseline to Post-manipulation in Study 2, across four groups. Asterisks denote significance between groups (difference between threat + efficacy and anger only groups). * *p* < .05, ** *p* < .01

A one-way ANOVA was conducted on the change statistics for each driving outcome. For the SPD, ANOVA results indicated that there were significant differences across groups [*F* (3, 80) = 2.86, *p =* .04, partial η^2^ = .10]. *Post-hoc* Tukey tests suggested that the threat + efficacy group (*M = −*8127.20, *SD* = 9208.72) differed from the anger only group (*M =* 839.05, *SD* = 10526.56; *p* = .03), and the direction of the change statistic suggested that the threat + efficacy group decreased speed while the anger only group increased speed. There were no other significant differences across groups (see Table 2 and Fig. 2).

**Table 2:** Descriptive Statistics for Driving Data Pre- and Post-Manipulation (Study 2)

	Threat + Efficacy + Anger	Threat + Efficacy	Anger Only	Control
	M (SD)	M (SD)	M (SD)	M (SD)
SPD (Pre)	42549.57 (13692.83)	46833.54 (9844.35)	42208.13 (8909.22)	40451.82 (11943.70)
SPD (Post)	40064.56 (14400.48)	38706.34 (9361.14)	43047.19 (11160.38)	35905.21 (11497.23)
SPD (Change)	−2485.02 (11588.32)	−8127.20 (9208.72)	839.05 (10526.56)	−4546.61 (7989.66)
Normal FOL (Pre)	12678.55 (3347.34)	13541.76 (4540.68)	13869.52 (3997.60)	13012.65 (4025.70)
Normal FOL (Post)	10864.83 (4329.12)	12537.92 (3968.86)	13301.15 (3910.92)	12634.27 (4368.02)
Normal FOL (Change)	−1813.73 (3844.65)	−1003.83 (3768.66)	−568.38 (2420.86)	−378.38 (2327.53)
Dangerous FOL (Pre)	18926.04 (3625.05)	19294.37 (2625.55)	19652.25 (3662.92)	19895.58 (4116.32)
Dangerous FOL (Post)	18133.03 (3074.06)	17964.00 (2763.55)	19127.21 (2728.67)	19273.11 (4473.25)
Dangerous FOL (Change)	−793.02 (2194.67)	−1330.37 (1768.65)	−525.04 (1356.78)	−622.47 (2053.02)
GAP (Pre)	8.15 (2.48)	8.20 (3.78)	7.75 (3.35)	9.25 (2.84)
GAP (Post)	8.60 (3.07)	7.75 (3.23)	9.21 (4.05)	9.70 (3.18)
GAP (Change)	.45 (2.50)	−.45 (1.76)	1.32 (1.92)	.45 (1.32)
OTK (Pre)	1.90 (.83)	2.20 (.89)	2.00 (.97)	2.00 (.92)
OTK (Post)	1.90 (.77)	1.80 (.89)	2.35 (.88)	2.00 (.73)
OTK (Change)	.00 (.63)	−.40 (1.14)	.35 (.67)	.00 (.86)

For Normal FOL change scores, because skew values indicated negative skew (= − .87), and since the K-S normality test was significant (*p* < .001), data were reflected and subjected to a log transformation. ANOVA results suggested that there were no overall significant differences across groups for either Normal FOL, *p* = .50, or Dangerous FOL, *p* = .55 (see Table 2).

**Fig. 3:**
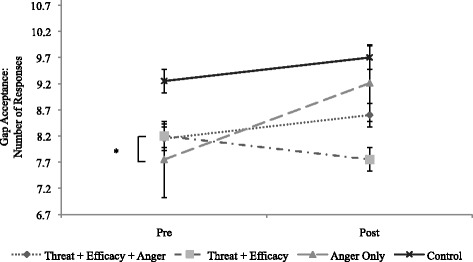
Change in Gap Acceptance from Baseline to Post-manipulation in Study 2, across four groups. Asterisks denote significance between groups (difference between threat + efficacy and anger only groups). * *p* < .05, ** *p* < .01

For GAP, ANOVA results revealed significant differences across groups, *F* (3, 78) = 2.75, *p <* .05, partial η^2^ = .01. *Post-hoc* Tukey tests indicated that the threat + efficacy group (*M = −*.45, *SD* = 1.76) differed significantly from the anger only group (*M =* 1.32, *SD* = 1.92; *p* = .03), with the direction of the change statistics indicating that the threat + efficacy group took fewer opportunities to emerge into traffic while the anger only group increased in the number of GAPs (see Table 2 and Fig. 3).

For the OTK scores, the overall difference across groups did not reach statistical significance (*p* = .06). However, since the ANOVA result approached significance, exploratory *post-hoc* Tukey test procedures were conducted. Findings suggested that the difference between the threat + efficacy (*M = −*.40, *SD* = 1.14) and the anger only (*M =* .35, *SD* = .67) groups was significant (*p* = .03), with the threat + efficacy group reducing their level of OTK and the anger only group displaying an increase in OTK (see Table 2).

## Discussion

Findings of Study 2 indicate that a high threat, high efficacy message can have an impact on a number of driving behaviours. The current findings also suggest that these effects may not be evident when individuals are under conditions of heightened emotional arousal (i.e. when they are angry). Specifically, while the threat + efficacy group evidenced a significant decrease, relative to the anger group, in three of the four risky driving measures, no such decrease was exhibited among the threat + efficacy + anger group. Previous research has found that anger provoked by one situation can lead to increased risk-seeking choices in a separate situation, while fear has the opposite effect [68]. In our study, participants who were exposed to the threat message *and* the anger manipulation (i.e. the threat + efficacy + anger group) did not change their driving behaviours relative to the other groups. This is an important finding because it suggests that, while threat appeals have the potential to be effective in moderating driving (as outlined in the EPPM), their effects may be diluted by the experience of anger, and potentially other emotions.

Anger is among the most commonly cited reasons for risky driving [72, 73], and previous research has made a strong case for how trait and state anger affect risky driving behaviours [22, 64, 72, 74]. In terms of its direct impact on driving, the anger findings from the current study are in line with previous research [22], indicating that participants who are experiencing state anger are likely to make more risky driving choices.

## General discussion

Despite disquiet about the effectiveness of threat appeals in health communication, their use remains common in road safety advertising campaigns. In part, this may be because research has not always reflected the multifaceted nature of such messages, nor the complexity of the psychological responses they trigger. Researchers have suggested that experimental studies examining the impact of these messages on driving behaviour are key to advancing our knowledge in the area [52, 75], but relatively few such studies have been conducted to date [76].

Our first experiment, informed by the EPPM, suggested that threat appeals are most likely to be effective in changing driving behaviour when combined with messages that target perceived efficacy. In this study, the threat + efficacy group selected significantly lower speeds at post-manipulation than the two groups who viewed the neutral advertisement. Participants in the threat only group did not differ significantly from the other three groups. Our second experiment sought to understand if, and how, state emotions can influence the impact of a threat appeal on driving behaviours. Anger was selected as a ‘proof of principle’ emotion, based on the correlational findings from Study 1, and on broader research linking anger to risky driving [22]. The results were as hypothesised, suggesting that state anger may counteract the potential value of combining fear-arousing threats and efficacy-building messages.

The exact psychological mechanisms underpinning this process are not entirely clear, but there are a number of plausible explanations. First, research into risk perception has suggested that anger leads to a more optimistic assessment of future events compared to fear [68, 77]. Crash-risk optimism has been identified as a cognitive bias that has a potentially maladaptive impact on driving behaviour [78], and risk perception is considered to be an important predictor of risky driving among young males [79]. Thus, if anger impacts negatively on risk perception, it may erode the effectiveness of a threatening communication.

Second, from an evolutionary perspective, fear and anger serve distinct functions [80], with anger serving to prepare us for combative situations – situations in which fear can erode our performance. Anger can make an individual’s attention “specific and targeted”, and can lead them to engage in “aggressive action” ([81]; p. 694]). This has applied relevance where threat appeals inadvertently elicit anger as well as fear. Research suggests that threat-based messages often elicit emotions other than those intended, or targeted, and that anger has adverse effects when aroused unintentionally or ‘collaterally’ ([82]; p.5, [83]).

Researchers have recently begun to examine in more detail the ‘emotional shift’ that occurs over the course of a health communication, and to consider the potential for these shifts to enhance the persuasion process [84]. It is clear from our research that focusing on individual emotions in persuasive communication studies has limited value, and that the array of affective responses generated by threat appeals, and those experienced following their exposure, needs to be probed more fully in experimental studies.

There are a number of strengths to this work. First, our initial physiological research demonstrates that, while threat appeal experiments operate on the premise that their manipulations arouse fear in the audience, this is not automatically achieved. For example, the ‘Boy’ road safety advertisement did not elicit more fear than the neutral advertisement, despite being rated as threatening in a pilot among a group of young people. This calls into question past research on threat appeals where the advertisements used have not been objectively verified to elicit fear. More generally, this highlights the importance of integrating, and accounting for, affective processes in theoretical and experimental threat appeal research.

Second, by examining the impact of a threatening road safety advertisement on a behaviour-based dependent variable, this research advances on previous studies in the area, which have predominantly relied on self-report driving outcomes. Driving behaviour is difficult to operationalise, and psychological studies have varied substantially in the measures they employ [85]. Self-report measures of driving are popular across studies of traffic and transport psychology [86], since valid self-report measures are relatively easy to use and cost-effective. However, the reliability and cross-cultural applicability of self-report measures of driving have been questioned [87]. Where one has limited access to driving simulators or in-vehicle data recorders, the type of computer-based driving task used in Studies 1 and 2 can be a cost-effective, culturally-specific alternative.

Following on from recommendations for researchers to use discrete dependent variables when measuring risky driving [60, 62, 88], we assessed the impact of a threat appeal on four distinct driving indices. The manipulation used in Study 2 effectively reduced risky driving in some behaviours (i.e. speeding) and not others (i.e. close following). Thus, we would conclude that it is overly-simplistic to use the umbrella term “risky driving behaviour” for all types of driver risk-taking. Just as it is difficult to generalise the effects of threat appeals from one health-risk behaviour to another, it is also problematic to apply conclusions from one type of risky driving to another. When examining the impact of various forms of persuasive communications on risky driving, it is becoming increasingly important for researchers to draw behaviour-specific conclusions and recommendations.

Third, current theoretical perspectives on threat appeals emphasise the roles of perceived threat and perceived efficacy [12] - constructs that are at the core of the EPPM [21]. However, the application of these general threat appeal frameworks to the area of road safety has been problematic, owing to a lack of controlled experimental studies in the area. In this research, we found perceptions of threat and efficacy to play an important role in shaping behavioural responses to a threat-based road safety advertisement. In Study 1, for example, while both groups who viewed the threat appeal had high levels of perceived threat, only the group with high perceptions of efficacy exhibited a change in speed choice.

Finally, while the literature has long highlighted the influence of anger on driving behaviour [22, 54, 63, 64, 72, 73, 89, 90], there has been little clarity surrounding the role played by state anger in moderating behavioural responses to a threat appeal message. In this research, the group who were presented with an anger-provoking task, in addition to the threat appeal message, did not change their driving behaviour from pre- to post-manipulation. Thus, while we have demonstrated the usefulness of the EPPM as a theoretical model through which to understand threat-based road safety advertisements, our findings also highlight the importance of controlling for state emotions, which may be affecting driving behaviour in real-life settings, but do not tend to be measured in the laboratory.

## Limitations and future directions

The present research contributes to a body of knowledge in the threat appeal and driving literature. However, a number of methodological limitations should be noted. First, the studies reported here utilised sample sizes based on medium effect sizes. This left us sufficiently powered to test differences between threat appeal and neutral conditions, but we may have been underpowered to detect differences across the threat appeal conditions. For example, while Study 1 findings supported our *a-priori* hypothesis that a threat + efficacy message would lead to a reduction in speed, there was no statistically significant difference between the threat + efficacy and threat only groups. Descriptive statistics indicated that the threat + efficacy group selected slower speeds, but the study may have been under-powered to detect significant effects between these two groups.

Second, the research did not deal with individual differences, despite research indicating that threat appeals can impact on males and females differentially [7, 91], and that personality can influence receptivity to messages by enhancing or eroding message efficacy [92]. Research has linked personality to risk-taking in general [93], and driver risk-taking in particular [74, 94], but this was not explored in the current research. Further, it is clear from the descriptive data that some within-group variability is present. We used randomisation to conditions and a change statistic to attempt to mitigate the effects of within-group variability, however, it was outside the scope of the current research to explore between- and within-group variability in detail. A more thorough exploration of variability in terms of individual differences, allowing such differences to be statistically controlled for, would be a valuable avenue for future research.

Third, the studies presented here focused on the immediate (i.e. short-term) impact of a threatening road safety advertisement, and we did not measure enduring effects. This has been a common feature of research in the area, and undermines the external validity of experimental research, particularly since such intervention effects can take time to manifest themselves [95]. In reality, audiences rarely get into their cars immediately after being exposed to road safety advertisements, and, while it is assumed that there will be a latent and enduring effect, there is a need to test this more rigorously. There is some evidence to suggest that, while threat-based messages are more persuasive in the short-term, positive (i.e. humorous) messages may be more effective in the long-term [96]. Additionally, since our aim was to test the conditions under which threat appeals are effective, we did not set out to test the effects of multiple advertisements. Future research could explore the generalisability of findings from this particular message to other types of threat-based approaches.

Fourth, the current research took place in Ireland, and focused on driving outcomes among a specific population of drivers. The manipulation used in this research changed driving behaviour on some indices (e.g. speed choice) but not others (e.g. close following), highlighting the importance of researchers in this area drawing behaviour-specific conclusions. Thus, while our findings are of theoretical relevance to the fields of public health and health communication broadly, further research is needed to examine the extent to which they are generalisable to other behaviours, populations and contexts.

Finally, while our VST and other bespoke dependent variables are strengths of the research, as with any laboratory-based driving measure, they cannot fully replicate real-world driving and ultimately lack ecological validity [97, 98].

Future research should address these limitations, and further probe the potential for affective states to enhance or undermine the efficacy of a threat-appeal message. As pointed out by Nabi [84], if we conceptualise human behaviour as being primarily influenced by emotion, then it is imperative that health communication research explores the full range and complexity of affective responses consistently, comprehensively and objectively. A key direction for future research into the effectiveness of public health campaigns concerns the identification of emotions that enhance, and those that hinder, the persuasive process.

## Conclusions

Road traffic collisions present a major social and financial challenge, and road safety has become a critical international public health issue. Prior to this research, a systematic examination of the conditions under which threat-based road safety advertisements are effective was lacking. By examining the roles of threat and efficacy in this context, the research contributes to the driving-specific evidence-base, as well as to the public health literature more generally. Findings highlight the importance of (i) including efficacy-building messages in threatening road safety advertising campaigns and (ii) integrating, and accounting for, state emotional variables in theoretical and experimental threat appeal research.

## Abbreviations

EMG, electromyography; EPPM, extended parallel process model; HBM, health belief model; HR, heart-rate; PMT, protection motivation theory; PRM, parallel response model; RTC, road traffic collision; SCL, skin conductance level; SRV, speeding and rule violation; TMT, terror management theory; TPB, theory of planned behaviour; VST, video speed test

## Additional files

Additional file 1: Study 1 Experimental Procedure. (JPG 85 kb) Additional file 2: Study 2 Experimental Procedure. (JPG 100 kb)
