# Supplementary figures and images for: Threat appeals in health communication: messages that elicit fear and enhance perceived efficacy positively impact on young male drivers
Source: BMC Public Health. 2016 Jul 27;16:645. doi: 10.1186/s12889-016-3227-2 (PMC4962518; doi:10.1186/s12889-016-3227-2)

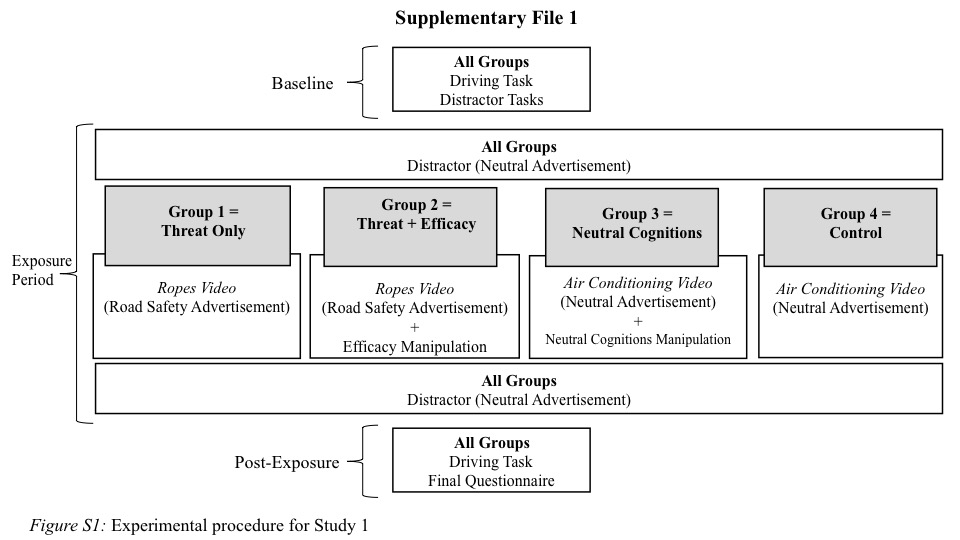

Supplement: Additional file 1: — Study 1 Experimental Procedure. (JPG 85 kb) [file 12889_2016_3227_MOESM1_ESM.jpg]

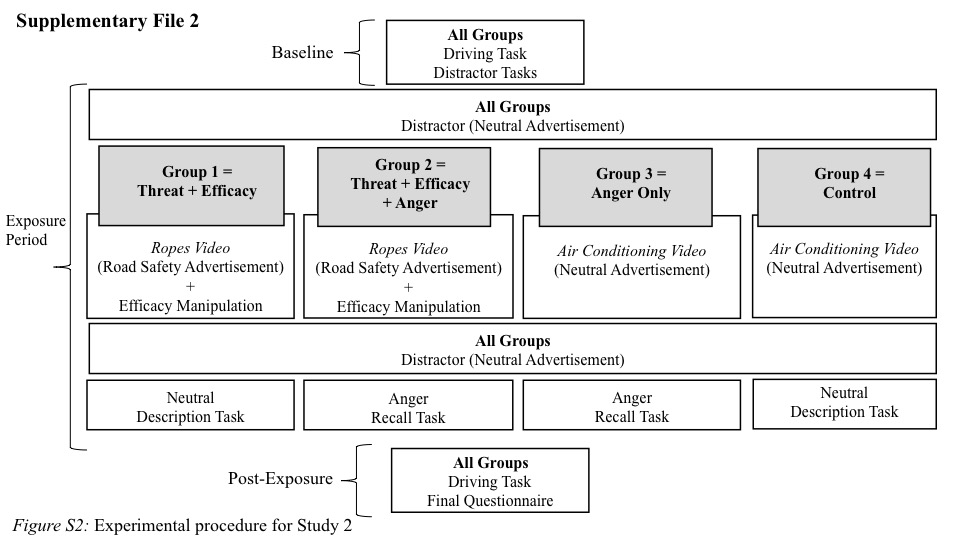

Supplement: Additional file 2: — Study 2 Experimental Procedure. (JPG 100 kb) [file 12889_2016_3227_MOESM2_ESM.jpg]
